# Supplementary material for: Synergistic Effect of Diet and Physical Activity on a NAFLD Cohort: Metabolomics Profile and Clinical Variable Evaluation
Source: Nutrients. 2023 May 25;15(11):2457. doi: 10.3390/nu15112457 (PMC10255762; doi:10.3390/nu15112457)
Supplement: Supplementary file 1 [file nutrients-15-02457-s001.zip › nutrients-2382063-supplementary.pdf]

Synergistic Effect of Diet and Physical Activity on a NAFLD Cohort: Metabolomics Profile and Clinical Variable Evaluation

Francesco Maria Calabrese [1], Giuseppe Celano [1,*], Caterina Bonfiglio [2], Angelo Campanella [2], Isabella Franco [2], Alessandro Annunziato [1], Gianluigi Giannelli [2], Alberto Ruben Osella [2,*,†] and Maria De Angelis [1,†]

1 Department of Soil, Plant and Food Science, University of Bari Aldo Moro, 70126 Bari, Italy; francesco.calabrese@uniba.it (F.M.C.); alessandro.annunziato@uniba.it (A.A.); maria.deangelis@uniba.it (M.D.A.)
2 National Institute of Gastroenterology S. De Bellis, IRCCS Research Hospital, Via Turi 27, 70013 Castellana Grotte, Italy; catia.bonfiglio@irccsdebellis.it (C.B.); angelo.campanella@irccsdebellis.it (A.C.); isabella.franco@irccsdebellis.it (I.F.); gianluigi.giannelli@irccsdebellis.it (G.G.)
* Correspondence: giuseppe.celano@uniba.it (G.C.); arosella@irccsdebellis.it (A.R.O.); Tel.: +39-0805442950 (G.C.); +39-0804994655 (A.R.O.)
† These authors equally contributed to the paper.

Supplementary Figure S1. Factor associated eigenvalues.
Scree plot reporting the eigenvalues for each one of the 28 identified factors resulting from biochemical clinical parameter measurements.

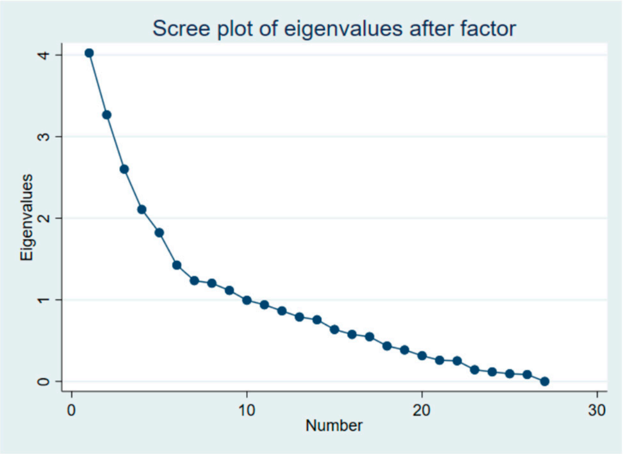

Supplementary Figure S2. MaAsLin2 associations between combined versus single intervention groups. Aerobic and non-aerobic physical activities (ATFIS), and the combination with Mediterranean diet intervention groups (LGIMD-ATFIS) were compared by means of the linear regression model (MaAsLin2), determining the multivariable associations between the phenotypes. Taxa relative abundances were reported on the Y axis. Only statistically significant association have been plotted.

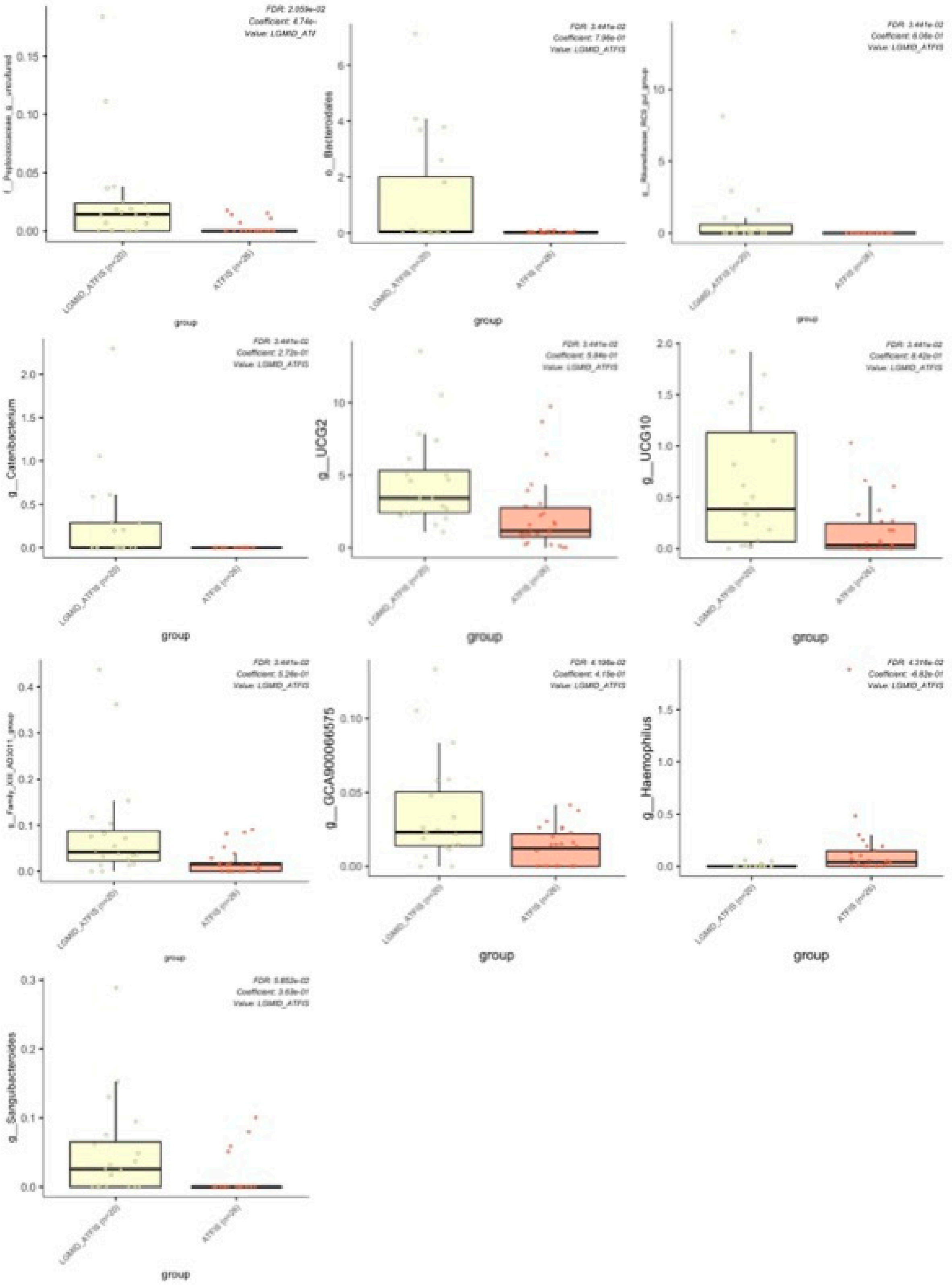

Supplementary Figure S3. Person correlation analysis between statistically significant VOCs and 16S rRNA annotated taxa. Annotated taxa from 16S rRNA sequencing experiment and volatile organic compounds that significantly differed in the ATFIS and LGIMD/ATFIS groups were correlated by means of a Pearson's test and only statically significant comparisons ($p<0.05$) were plotted. Black and red fonts mark biochemical parameters and VOCs, respectively. In the legend scale bar, green and purple colours are meaningful of positive and negative correlation, respectively. The cross-group paired variables were highlighted with red (positive) and green (negative) background colour, respectively.

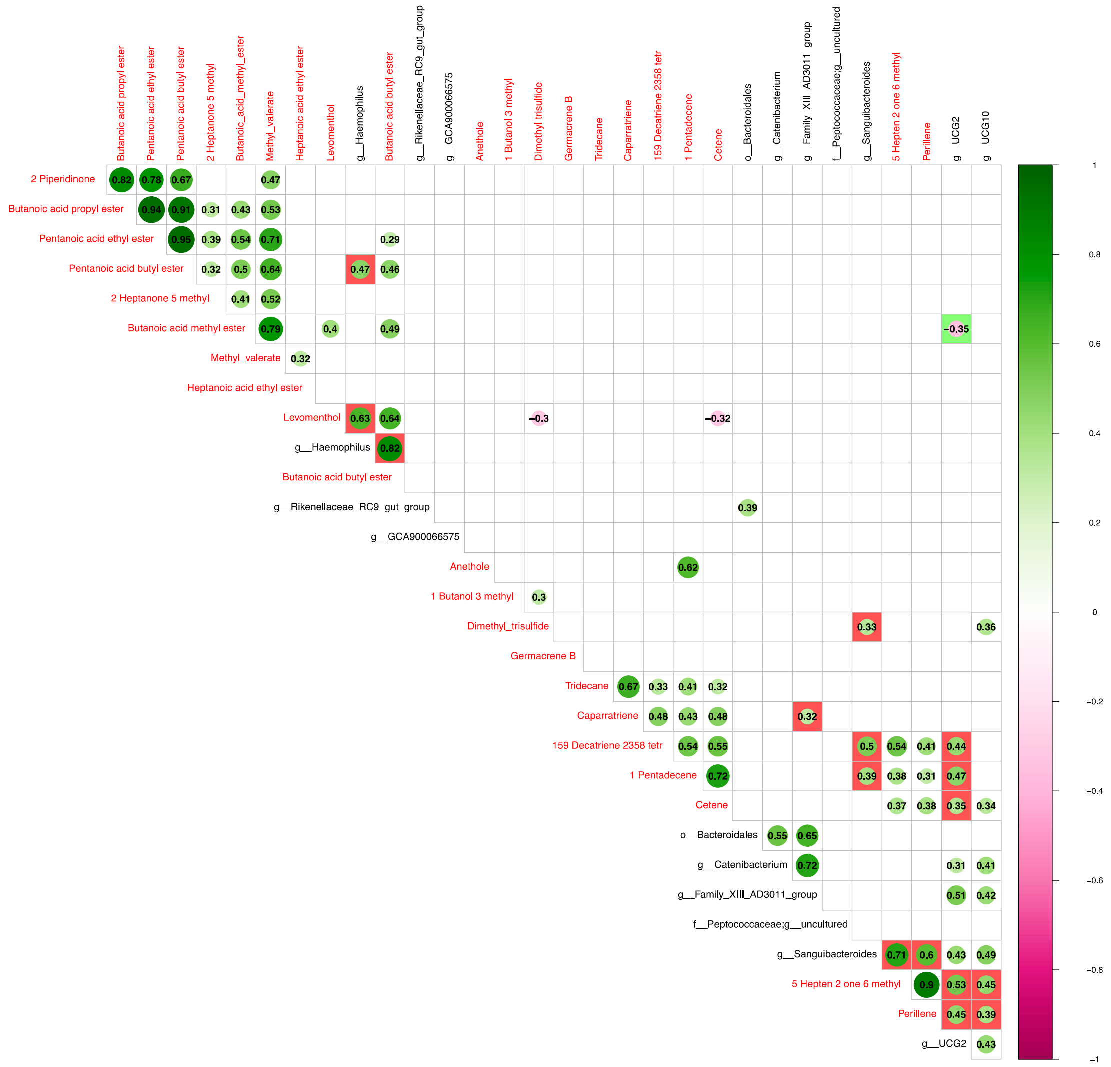

Table S1. **Complete panel of detected VOCs**. Concentration of volatile organic metabolites from an untargeted GC-MS metabolomics experiment.

**sample_id**	**20010_1**	**20010_2**	**20011_1**	**20011_2**	**20016_1**	**20016_2**	**20018_1**	**20018_2**	**20020_1**	**20020_2**
group	**LGIMD-ATFIS**	**LGIMD-ATFIS**	**LGIMD-ATFIS**	**LGIMD-ATFIS**	**LGIMD-ATFIS**	**LGIMD-ATFIS**	**LGIMD-ATFIS**	**LGIMD-ATFIS**	**LGIMD-ATFIS**	**LGIMD-ATFIS**
Methyl_acetate	0.000	0.000	0.000	0.000	0.000	0.000	0.000	0.000	0.000	0.000
Ethyl_Acetate	0.000	0.000	0.000	0.000	0.000	0.000	0.000	0.000	0.000	0.000

2-Butanone	0.060	0.297	0.044	0.081	0.302	1.128	0.018	0.040	0.181	0.216
Butanal_3-methyl-	0.761	1.705	0.580	0.598	3.987	3.477	1.462	0.963	2.095	1.262
Ethanol	0.175	0.111	0.146	0.102	0.113	0.159	0.000	0.000	0.000	0.035
Propanoic_acid_ethyl_ester	0.000	0.000	0.118	0.000	0.000	0.000	0.000	0.000	0.000	0.000
2-Pentanone	0.000	0.000	0.378	0.233	0.246	0.406	0.000	0.000	0.375	0.454
Butanoic_acid_methyl_ester	0.000	0.000	0.269	0.000	0.000	0.000	0.000	0.000	0.000	0.000
Methyl_Isobutyl_Ketone	0.286	0.259	0.039	0.049	0.082	0.062	0.052	0.059	0.086	0.057
a-Pinene	0.000	0.000	0.000	0.000	0.132	0.000	0.113	0.283	0.932	0.186
Disulfide_dimethyl	0.272	0.227	0.301	0.072	0.144	0.000	0.000	0.077	0.824	0.000
Butanoic_acid_3-methyl-_ethy	0.100	0.027	0.000	0.031	0.000	0.000	0.000	0.000	0.000	0.000
Methyl_valerate	0.000	0.000	0.096	0.000	0.000	0.000	0.000	0.000	0.000	0.000
b-Pinene	0.000	0.000	0.000	0.000	0.115	0.000	0.152	0.430	4.264	0.135
Butanoic_acid_propyl_ester	0.272	0.000	0.747	0.000	0.000	0.000	0.000	0.000	0.000	0.000
3-Carene	0.000	0.000	0.000	0.000	0.197	0.000	0.512	1.140	0.127	0.000
Pentanoic_acid_ethyl_ester	0.257	0.000	0.490	0.000	0.000	0.000	0.000	0.000	0.000	0.000
(2E4E)-37-Dimethylocta-24-d	0.000	0.000	0.000	0.129	0.000	0.000	0.157	0.149	0.000	0.000
a-Phellandrene	0.000	0.000	0.000	0.000	0.000	0.000	0.189	0.142	0.000	0.028
b-Myrcene	0.000	0.000	0.000	0.000	0.000	0.000	0.067	0.000	1.494	0.135
Isovaleric_acid_propyl_ester	0.000	0.000	0.000	0.000	0.000	0.000	0.000	0.000	0.000	0.000
Terpinolene	0.000	0.000	0.000	0.000	0.000	0.000	0.000	0.000	0.000	0.262
1-Butanol	0.664	0.000	1.317	0.348	0.000	0.000	0.024	0.000	0.000	0.000
D-Limonene	4.599	2.714	0.573	0.202	0.212	0.303	0.488	0.260	117.502	0.736
beta-Phellandrene	0.000	0.000	0.000	0.000	0.000	0.000	0.000	0.100	0.000	0.205
Butanoic_acid_butyl_ester	0.000	0.000	0.801	0.036	0.000	0.000	0.000	0.000	0.000	0.000
Pentanoic_acid_propyl_ester	0.000	0.000	0.314	0.000	0.000	0.000	0.000	0.000	0.000	0.000
g-Terpinene	0.307	0.000	0.000	0.000	0.000	0.000	0.045	0.161	6.227	0.917
Hexanoic_acid_ethyl_ester	0.091	0.000	1.106	0.109	0.000	0.000	0.000	0.000	0.000	0.085
1-Butanol_3-methyl-	0.253	0.000	0.000	0.000	0.125	0.000	0.000	0.086	0.115	0.000
b-Ocimene	0.000	0.000	0.000	0.000	0.058	0.000	0.000	0.000	0.195	0.002
Styrene	0.000	0.000	0.000	0.114	0.000	0.000	0.029	0.000	0.000	0.000
p-Cymene	0.000	0.000	0.000	0.000	0.000	0.237	0.031	0.193	0.526	0.908
2-Heptanone_5-methyl-	0.000	0.000	0.000	0.000	0.090	0.000	0.023	0.000	0.000	0.000
2-Carene	0.000	0.000	0.000	0.000	0.000	0.000	0.000	0.000	0.305	0.000
1-Pentanol	0.192	0.000	0.319	0.153	0.000	0.000	0.030	0.000	0.108	0.000
Acetic_acid_hexyl_ester	0.000	0.000	0.000	0.000	0.000	0.000	0.000	0.000	0.000	0.000
Pentanoic_acid_4-methyl-_pen	0.000	0.000	0.000	0.000	0.000	0.000	0.000	0.000	0.000	0.000
Octanal	0.116	0.000	0.000	0.000	0.000	0.000	0.000	0.000	0.000	0.000
2-Octanone	0.000	0.000	0.068	0.124	0.251	0.055	0.000	0.000	0.438	0.214
Acetoin	0.000	0.000	0.000	0.000	0.079	0.000	0.000	0.000	0.000	0.000
Pentanoic_acid_butyl_ester	0.082	0.000	0.574	0.023	0.000	0.000	0.000	0.000	0.000	0.000

Butanoic_acid_pentyl_ester	0.181	0.000	0.000	0.000	0.000	0.000	0.000	0.000	0.000	0.000
Hexanoic_acid_propyl_ester	0.000	0.000	0.638	0.037	0.000	0.000	0.000	0.000	0.000	0.032
Tridecane	0.000	0.000	0.000	0.038	0.054	0.011	0.000	0.026	0.065	0.000
5-Hepten-2-one_6-methyl-	0.000	6.294	10.022	9.080	15.438	92.711	4.963	4.082	2.421	5.000
Heptanoic_acid_ethyl_ester	0.000	0.000	0.000	0.000	0.000	0.000	0.000	0.000	0.000	0.000
Dimethyl_trisulfide	0.286	0.000	0.495	0.000	0.000	0.000	0.000	0.000	0.411	0.000
Allyl_Isothiocyanate	0.000	0.000	0.000	0.000	0.000	0.000	0.000	0.000	0.000	0.000
2-Hydroxy-3-pentanone	0.000	0.000	0.000	0.000	0.000	0.000	0.000	0.000	0.000	0.000
Hexanoic_acid_isobutyl_ester	0.000	0.000	0.000	0.000	0.000	0.000	0.000	0.000	0.000	0.000
2-butyl-1-octanol	0.096	0.148	0.000	0.000	0.001	0.444	0.055	0.007	0.054	0.121
Cyclohexanecarboxylic_acid_me	0.000	0.000	0.120	0.051	0.000	0.000	0.021	0.000	0.000	0.000
1-Hexanol	0.000	0.000	0.734	0.419	0.000	0.000	0.000	0.000	0.081	0.392
Nonanal	0.332	0.058	0.241	0.464	0.000	0.329	0.272	0.333	0.396	0.604
2-Nonanone	0.000	0.000	0.000	0.000	0.172	0.148	0.000	0.000	0.000	0.529
Cyclohexanecarboxylic_acid_et	0.000	0.000	0.000	0.525	0.000	0.000	0.020	0.000	0.000	0.000
Perillene	0.000	0.045	0.363	0.000	0.114	0.693	0.088	0.038	0.026	0.083
Pentanoic_acid_pentyl_ester	0.146	0.000	0.000	0.000	0.000	0.000	0.000	0.000	0.000	0.000
Hexanoic_acid_butyl_ester	0.000	0.000	0.826	0.107	0.000	0.000	0.000	0.000	0.000	0.000
Butanoic_acid_hexyl_ester	0.042	0.000	0.386	0.084	0.000	0.000	0.000	0.000	0.000	0.032
Tetradecane	0.889	0.662	0.141	0.084	0.779	0.878	0.000	0.661	0.877	0.127
Octanoic_acid_ethyl_ester	0.000	0.000	0.000	0.000	0.000	0.000	0.000	0.000	0.000	0.000
Acetic_acid	11.532	1.336	23.532	8.546	2.523	2.211	1.351	2.086	4.993	2.073
(+)-2-Bornanone	0.000	0.000	0.000	0.000	0.000	0.000	0.000	0.000	0.000	0.000
2-Decanone	0.000	0.000	0.000	0.000	0.032	0.037	0.003	0.004	0.000	0.017
Benzaldehyde	0.957	3.272	0.755	1.278	2.198	3.887	1.099	1.042	0.915	1.622
1-Hexanol_2-ethyl-	0.000	0.138	0.000	0.000	0.000	0.000	0.000	0.064	0.000	0.000
2-Nonenal_(E)-	0.112	0.007	0.156	0.117	0.000	0.060	0.212	0.105	0.078	0.095
Pentadecane	0.000	0.815	0.000	0.000	0.000	0.760	0.000	0.000	0.000	0.000
Propanoic_acid	9.552	1.158	8.681	5.844	2.030	0.565	0.956	1.233	4.163	1.307
Caparratriene	0.000	0.942	0.000	0.000	0.395	0.415	0.055	0.089	0.000	0.000
1-Pentadecene	0.238	0.599	0.100	0.077	1.843	1.321	0.000	0.021	2.297	0.010
Linalool	0.000	0.000	0.185	0.505	0.000	0.000	0.256	0.466	0.000	1.403
Propanoic_acid_2-methyl-	4.348	1.134	1.835	1.907	0.000	0.000	0.229	0.361	3.995	0.818
Caryophyllene	0.509	0.000	0.037	0.262	10.602	0.409	13.265	2.571	2.572	0.282
Methyl_carvacrol	0.000	0.000	0.000	0.000	0.000	0.000	0.000	0.000	0.000	0.467
Dichloroacetic_acid_4-pentade	0.000	0.000	0.000	0.000	0.000	0.000	0.000	0.000	0.613	0.000
2-Undecanone	0.545	1.269	0.182	0.494	1.561	3.373	0.733	0.905	0.551	0.000
159-Decatriene_2358-tetr	0.000	0.521	0.000	0.000	0.583	0.929	0.000	0.000	0.283	0.000
Benzeneacetaldehyde	0.000	0.000	0.000	0.000	0.000	0.000	0.000	0.000	0.000	0.000

Butanoic_acid	39.256	6.374	73.989	35.601	5.162	2.267	3.079	3.375	10.900	10.989
Hexadecane	2.475	3.880	0.386	0.286	0.566	0.384	0.000	0.106	0.482	0.148
Levomenthol	0.000	0.163	0.000	0.000	0.000	0.322	0.357	0.000	0.000	0.808
Hexanoic_acid_2-methyl-	22.643	10.008	8.853	10.439	6.142	5.901	1.264	1.727	13.713	5.455
Cetene	0.081	0.219	0.370	0.000	0.660	0.337	0.000	0.000	0.281	0.000
Valencene	1.623	0.769	7.124	0.000	1.281	0.607	1.122	0.095	1.978	0.000
a-Muurolene	0.411	0.000	0.000	0.000	0.350	0.000	0.000	0.000	0.000	0.000
b-Bisabolene	0.000	0.000	0.082	0.000	0.138	0.044	0.158	0.000	2.900	0.024
Nonadecane	0.000	1.408	0.000	0.000	0.000	0.000	0.000	0.000	0.000	0.000
Pentanoic_acid	0.087	4.873	20.424	16.074	2.743	2.255	1.340	1.563	6.958	4.782
g-Cadinene	0.000	0.000	0.000	0.000	0.734	0.000	0.194	0.175	0.000	0.000
trans-g-Bisabolene	0.293	0.000	0.000	0.857	0.781	0.569	0.755	0.000	3.599	0.000
trans-a-Bisabolene	0.000	0.000	0.000	0.000	0.000	0.000	0.000	0.000	0.343	0.000
Aromandendrene	0.000	0.000	0.081	0.215	0.000	0.000	0.070	0.000	0.000	0.000
Pentanoic_acid_4-methyl-	0.362	0.000	0.905	0.542	0.007	0.043	0.050	0.047	0.072	0.184
Germacrene_B	0.000	0.000	0.000	0.000	0.147	0.000	0.000	0.000	0.000	0.000
Anethole	1.909	0.715	0.000	0.126	0.109	0.857	0.193	0.159	21.056	0.056
2-Tridecanone	0.332	0.695	0.373	0.297	0.447	0.738	0.216	0.363	0.000	0.132
Octadecane	1.011	4.935	0.000	0.000	0.022	0.000	0.000	0.000	0.040	0.000
Hexanoic_acid	4.859	0.000	62.256	37.512	4.204	1.798	0.583	0.384	6.151	26.975
59-Undecadien-2-one_610-dim	0.065	0.000	0.641	0.512	0.237	0.947	0.248	0.125	0.237	0.329
2-Tetradecanone	0.277	0.254	0.000	0.158	0.180	0.000	0.053	0.061	0.220	0.113
Phenylethyl_Alcohol	0.852	0.254	0.697	0.701	0.000	0.000	0.000	0.000	0.897	0.439
2-Tetradecanone	0.279	0.360	0.000	0.000	0.239	0.108	0.000	0.000	0.045	0.000
Heptanoic_acid	0.000	0.000	8.713	10.681	2.996	0.645	0.136	0.169	3.091	6.149
Phenol	2.246	1.196	3.701	2.047	0.105	0.140	4.212	1.550	0.108	0.146
2-Pentadecanone	0.463	1.910	0.388	0.335	0.964	0.975	0.158	0.139	0.311	0.101
3-Phenylpropanol	0.396	0.000	0.346	0.457	0.000	0.000	0.000	0.000	0.000	0.135
Octanoic_acid	0.185	0.000	2.403	2.764	0.252	0.906	0.122	0.192	0.520	2.080
Phenol_2-methyl-	53.746	89.820	39.749	49.591	100.830	66.807	44.949	38.012	82.591	38.855
2-Hexadecanone	0.368	1.154	0.000	0.000	0.283	0.178	0.000	0.000	0.000	0.000
2-Piperidinone	0.000	0.000	0.000	0.000	0.000	0.000	0.000	0.000	0.000	0.000
2-Tridecanol	0.257	1.123	0.275	0.176	0.253	0.000	0.000	0.000	0.772	0.000
Nonanoic_acid	0.475	0.800	2.926	7.390	9.147	8.303	6.488	7.523	2.851	6.692
Carvacrol	0.000	0.000	0.000	0.000	0.000	0.000	0.000	0.000	0.000	0.562
Ethanone_1-(2-aminophenyl)-	0.000	0.000	0.000	0.000	0.724	0.599	0.045	0.121	0.000	0.000
1H-Pyrrole-25-dione_3-ethyl-	0.293	0.926	0.111	0.160	0.153	0.316	0.569	0.302	0.202	0.073
1-Tetracosene	0.076	1.481	0.172	0.097	0.210	0.061	0.000	0.000	0.482	0.028
n-Decanoic_acid	0.315	0.109	0.498	0.833	0.325	0.604	0.096	0.000	0.482	0.314
1-Hexadecanol	1.972	5.940	6.229	2.950	0.824	0.542	0.004	0.000	2.246	0.543
(Z)6-Pentadecen-1-ol	0.209	0.318	0.448	0.178	0.000	0.000	0.448	0.000	0.175	0.040
g-Dodecalactone	0.465	0.374	0.473	0.453	0.269	0.889	0.552	0.393	0.221	0.104
Indole	15.395	18.224	5.476	2.388	21.637	10.690	18.943	22.614	22.141	7.089
1H-Indole_5-methyl-	0.500	0.000	6.407	5.411	0.000	29.426	1.682	6.832	22.853	0.000
Benzeneacetic_acid	0.245	0.144	0.370	0.514	0.095	0.205	0.089	0.134	0.000	0.181
Benzenepropanoic_acid_silver(	0.587	0.219	2.689	1.680	0.000	0.171	0.363	0.094	0.395	0.345

2H-Indol-2-one_13-dihydro-	0.143	0.315	0.092	0.160	0.240	0.164	0.057	0.062	0.101	0.055
**sample_id**	**20029_1**	**20029_2**	**20030_1**	**20030_2**	**20038_1**	**20038_2**	**20043_1**	**20043_2**	**20052_1**	**20052_2**
**group**	**LGIMD-ATFIS**	**LGIMD-ATFIS**	**LGIMD-ATFIS**	**LGIMD-ATFIS**	**LGIMD-ATFIS**	**LGIMD-ATFIS**	**LGIMD-ATFIS**	**LGIMD-ATFIS**	**ATFIS**	**ATFIS**
Methyl_acetate	0.000	0.000	0.000	0.000	0.000	0.000	0.000	0.000	0.000	0.000
Ethyl_Acetate	0.000	0.000	0.000	0.000	0.000	0.000	0.000	0.000	0.000	0.000
2-Butanone	0.034	0.171	0.063	0.054	0.020	0.045	0.067	0.044	0.000	0.101
Butanal_3-methyl-	0.867	1.562	1.602	2.869	1.524	2.794	2.613	3.212	3.179	0.637
Ethanol	0.069	0.072	0.000	0.000	0.000	0.000	0.139	0.064	0.000	0.032
Propanoic_acid_ethyl_ester	0.000	0.000	0.000	0.000	0.000	0.000	0.000	0.000	0.000	0.000
2-Pentanone	0.000	0.358	0.000	0.000	0.000	0.305	0.269	0.205	0.444	1.158
Butanoic_acid_methyl_ester	0.000	0.000	0.000	0.000	0.000	0.000	0.000	0.000	0.000	0.000
Methyl_Isobutyl_Ketone	0.076	0.151	0.274	0.261	0.068	0.050	0.088	0.068	0.145	0.233
a-Pinene	0.000	0.000	0.127	0.037	0.048	0.157	0.129	0.167	0.000	0.000
Disulfide_dimethyl	0.315	0.000	1.724	0.800	0.450	0.507	0.500	0.249	0.778	0.402
Butanoic_acid_3-methyl-_ethy	0.000	0.000	0.000	0.000	0.000	0.000	0.042	0.008	0.000	0.000
Methyl_valerate	0.000	0.000	0.000	0.000	0.000	0.000	0.000	0.000	0.000	0.000
b-Pinene	0.000	0.000	0.709	0.000	0.000	0.096	0.213	0.000	0.000	0.000
Butanoic_acid_propyl_ester	0.000	0.000	0.000	0.000	0.000	0.000	0.000	0.000	0.000	0.000
3-Carene	0.000	0.000	0.000	0.000	0.000	0.730	0.000	0.181	0.000	0.000
Pentanoic_acid_ethyl_ester	0.000	0.000	0.000	0.000	0.000	0.000	0.000	0.000	0.000	0.000
(2E4E)-37-Dimethylocta-24-d	0.215	0.462	0.000	0.000	0.000	0.000	0.000	0.000	0.170	0.117
a-Phellandrene	0.000	0.000	0.385	0.000	0.000	0.034	0.000	0.000	0.000	0.000
b-Myrcene	0.000	0.000	0.000	0.173	0.000	0.134	0.000	0.000	0.000	0.000
Isovaleric_acid_propyl_ester	0.000	0.000	0.000	0.000	0.000	0.000	0.000	0.000	0.000	0.000
Terpinolene	0.000	0.000	0.097	0.447	0.000	0.130	0.000	0.000	0.000	0.000
1-Butanol	0.000	0.000	0.000	0.000	0.000	0.000	0.000	0.146	0.000	0.173
D-Limonene	0.629	0.317	0.929	0.252	0.308	2.791	2.578	0.285	3.200	0.244
beta-Phellandrene	0.000	0.000	0.000	0.000	0.000	0.000	0.000	0.000	0.000	0.000
Butanoic_acid_butyl_ester	0.000	0.028	0.037	0.000	0.000	0.000	0.000	0.000	0.000	0.000
Pentanoic_acid_propyl_ester	0.000	0.000	0.000	0.000	0.000	0.000	0.000	0.000	0.000	0.000
g-Terpinene	0.000	0.000	1.452	0.016	0.034	1.184	0.189	0.307	0.000	0.051
Hexanoic_acid_ethyl_ester	0.022	0.082	0.000	0.000	0.026	0.000	0.000	0.000	0.000	0.028
1-Butanol_3-methyl-	0.136	0.128	0.176	0.218	0.071	0.000	0.182	0.347	0.000	0.062
b-Ocimene	0.000	0.000	0.111	0.000	0.000	0.003	0.000	0.000	0.000	0.000
Styrene	0.000	0.000	0.000	0.000	0.000	0.000	0.000	0.068	0.000	0.000
p-Cymene	0.000	0.000	0.376	0.238	0.000	0.774	0.078	0.220	0.000	0.000
2-Heptanone_5-methyl-	0.064	0.000	0.000	0.000	0.069	0.000	0.000	0.000	0.055	0.109
2-Carene	0.000	0.000	0.149	0.000	0.000	0.000	0.080	0.000	0.000	0.052
1-Pentanol	0.111	0.000	0.111	0.058	0.000	0.000	0.077	0.151	0.000	0.061

Acetic_acid_hexyl_ester	0.000	0.000	0.000	0.000	0.000	0.000	0.000	0.000	0.000	0.000
Pentanoic_acid_4-methyl-_pen	0.000	0.000	0.000	0.000	0.000	0.000	0.000	0.000	0.000	0.000
Octanal	0.000	0.000	0.000	0.000	0.000	0.000	0.000	0.000	0.000	0.000
2-Octanone	0.069	0.164	0.137	0.125	0.242	0.000	0.163	0.202	0.268	0.135
Acetoin	0.368	0.000	0.000	0.000	0.000	0.249	0.000	0.139	0.000	4.522
Pentanoic_acid_butyl_ester	0.000	0.000	0.096	0.000	0.000	0.000	0.000	0.000	0.000	0.000
Butanoic_acid_pentyl_ester	0.000	0.000	0.000	0.000	0.000	0.000	0.000	0.000	0.000	0.000
Hexanoic_acid_propyl_ester	0.000	0.000	0.059	0.000	0.000	0.000	0.000	0.000	0.000	0.022
Tridecane	0.031	0.000	0.004	0.011	0.048	0.000	0.335	0.003	0.000	0.000
5-Hepten-2-one_6-methyl-	7.703	2.123	9.610	4.272	3.806	3.883	4.295	5.775	2.952	0.766
Heptanoic_acid_ethyl_ester	0.000	0.000	0.000	0.000	0.000	0.000	0.000	0.000	0.000	0.000
Dimethyl_trisulfide	0.176	0.000	0.948	0.258	0.291	0.224	0.133	0.000	0.269	0.145
Allyl_Isothiocyanate	0.000	0.000	0.000	0.000	0.000	0.000	0.000	0.000	0.000	0.000
2-Hydroxy-3-pentanone	0.019	0.000	0.000	0.000	0.000	0.000	0.000	0.042	0.102	0.000
Hexanoic_acid_isobutyl_ester	0.000	0.000	0.036	0.000	0.008	0.000	0.000	0.000	0.000	0.000
2-butyl-1-octanol	0.022	0.074	0.001	0.043	0.000	0.074	0.000	0.000	0.039	0.000
Cyclohexanecarboxylic_acid_me	0.020	0.000	0.000	0.000	0.047	0.000	0.000	0.000	0.000	0.033
1-Hexanol	0.141	0.000	0.255	0.078	0.151	0.000	0.136	0.500	0.143	0.435
Nonanal	0.124	0.855	0.494	0.283	0.352	0.192	0.280	0.201	0.406	0.368
2-Nonanone	0.037	0.000	0.000	0.000	0.000	0.000	0.000	0.112	0.000	0.171
Cyclohexanecarboxylic_acid_et	0.000	0.000	0.000	0.000	0.062	0.000	0.000	0.000	0.000	0.000
Perillene	0.114	0.028	0.055	0.000	0.099	0.048	0.000	0.000	0.045	0.000
Pentanoic_acid_pentyl_ester	0.000	0.000	0.000	0.000	0.000	0.000	0.000	0.000	0.000	0.000
Hexanoic_acid_butyl_ester	0.000	0.000	0.184	0.000	0.000	0.000	0.000	0.000	0.000	0.000
Butanoic_acid_hexyl_ester	0.000	0.000	0.060	0.000	0.000	0.000	0.000	0.000	0.000	0.029
Tetradecane	0.356	0.243	0.515	0.514	0.332	0.596	0.152	0.000	0.419	0.000
Octanoic_acid_ethyl_ester	0.000	0.000	0.000	0.000	0.000	0.000	0.000	0.000	0.000	0.000
Acetic_acid	5.513	11.692	3.505	1.434	7.096	1.210	5.073	3.637	1.962	4.021
(+)-2-Bornanone	0.000	0.000	0.000	0.000	0.000	0.000	0.000	0.000	0.000	0.000
2-Decanone	0.004	0.000	0.000	0.000	0.000	0.000	0.043	0.000	0.000	0.000
Benzaldehyde	1.564	1.600	1.162	1.803	0.993	2.496	0.977	2.244	1.665	0.484
1-Hexanol_2-ethyl-	0.000	0.000	0.207	0.000	0.000	0.000	0.000	0.184	0.000	0.000
2-Nonenal_(E)-	0.068	0.211	0.107	0.001	0.060	0.060	0.062	0.056	0.000	0.085
Pentadecane	0.000	0.000	0.000	0.000	0.000	0.000	0.000	0.000	0.524	0.000
Propanoic_acid	4.995	9.411	2.801	1.385	3.595	1.368	4.465	2.493	0.583	2.213
Caparratriene	0.455	0.000	0.000	0.000	0.000	0.000	1.419	0.000	0.000	0.000
1-Pentadecene	0.177	0.524	0.186	0.443	0.510	0.295	1.236	0.000	0.475	0.244
Linalool	0.000	0.000	0.966	1.103	0.000	0.000	0.000	0.298	0.000	0.000
Propanoic_acid_2-methyl-	1.549	5.860	1.661	0.831	1.794	1.116	2.312	1.093	0.209	0.887

Caryophyllene	0.032	0.214	2.441	1.266	1.621	0.673	1.781	1.216	6.085	0.702
Methyl_carvacrol	0.000	0.000	0.000	2.151	0.000	0.787	0.000	0.012	0.000	0.000
Dichloroacetic_acid_4-pentade	0.142	0.084	0.000	0.000	0.422	0.000	0.387	0.000	0.000	0.000
2-Undecanone	0.501	0.208	0.286	0.329	0.000	0.351	0.739	0.994	0.986	0.644
159-Decatriene_2358-tetr	0.000	0.000	0.000	0.000	0.000	0.000	0.526	0.807	0.000	0.000
Benzeneacetaldehyde	0.000	0.000	0.368	0.997	0.000	0.000	0.000	0.000	0.693	0.099
Butanoic_acid	12.239	33.255	9.993	3.177	19.294	7.588	6.872	6.819	2.987	12.880
Hexadecane	0.331	0.000	0.276	0.272	0.172	0.272	0.471	0.448	0.362	0.231
Levomenthol	0.070	0.988	0.000	0.000	0.000	0.000	0.000	0.000	0.000	0.925
Hexanoic_acid_2-methyl-	6.684	27.506	7.572	4.516	7.324	6.161	7.577	4.426	2.292	4.323
Cetene	0.160	0.088	0.206	0.352	0.173	0.154	0.419	0.294	0.104	0.130
Valencene	0.000	0.000	0.338	0.934	0.358	0.000	0.623	0.000	0.193	0.209
a-Muurolene	0.000	0.000	0.000	0.000	0.000	0.000	0.000	0.000	0.000	0.000
b-Bisabolene	0.000	0.000	0.501	0.707	0.000	0.194	0.197	0.500	0.393	0.000
Nonadecane	0.000	0.000	0.000	0.000	0.000	0.000	0.000	0.000	0.000	0.000
Pentanoic_acid	9.108	18.704	5.191	2.435	11.730	5.966	4.642	5.048	1.934	7.590
g-Cadinene	0.000	0.000	0.000	0.533	0.000	0.120	0.000	0.000	0.583	0.000
trans-g-Bisabolene	0.000	0.000	1.000	0.424	0.255	0.000	1.934	7.721	0.386	0.316
trans-a-Bisabolene	0.000	0.000	0.093	0.055	0.000	0.000	0.000	0.000	0.174	0.000
Aromandendrene	0.058	0.000	0.000	0.000	0.000	0.000	0.000	0.000	0.000	0.000
Pentanoic_acid_4-methyl-	0.413	0.094	0.060	0.000	0.435	0.173	0.090	0.109	0.000	0.000
Germacrene_B	0.000	0.000	0.000	0.000	0.000	0.000	0.000	0.000	0.000	0.000
Anethole	0.946	0.117	2.128	0.332	0.470	0.420	0.116	0.073	0.131	0.466
2-Tridecanone	0.309	0.126	0.000	0.399	0.000	0.220	0.410	0.397	0.365	0.452
Octadecane	0.000	0.000	0.235	0.273	0.000	0.000	0.000	2.360	0.000	0.000
Hexanoic_acid	9.608	16.745	13.306	6.712	31.129	13.297	4.829	14.429	4.382	38.006
59-Undecadien-2-one_610-dim	0.217	0.233	0.351	0.576	0.412	0.291	0.336	0.239	0.959	0.321
2-Tetradecanone	0.219	0.000	0.000	0.302	0.000	0.214	0.178	0.000	0.000	0.000
Phenylethyl_Alcohol	0.485	0.401	0.453	0.420	0.000	0.000	0.000	0.000	0.437	0.488
2-Tetradecanone	0.200	0.000	0.051	0.084	0.000	0.000	0.089	0.124	0.051	0.077
Heptanoic_acid	4.676	4.998	4.835	2.707	19.376	7.490	2.871	7.918	1.911	15.721
Phenol	0.650	0.231	0.183	0.597	0.996	0.748	0.212	0.210	0.377	3.340
2-Pentadecanone	0.377	0.229	0.288	0.601	0.156	0.239	0.326	0.288	0.292	0.202
3-Phenylpropanol	0.106	0.000	0.111	0.101	0.000	0.000	0.000	0.000	0.036	0.160
Octanoic_acid	0.597	0.656	1.667	0.780	8.935	2.204	0.429	2.213	0.613	5.120
Phenol_2-methyl-	47.088	96.946	57.084	46.595	51.445	67.567	74.549	56.422	53.452	38.830
2-Hexadecanone	0.159	0.000	0.000	0.274	0.000	0.000	0.000	0.000	0.000	0.000
2-Piperidinone	0.000	0.000	0.000	0.000	0.000	0.000	0.000	0.000	0.171	0.326
2-Tridecanol	0.260	0.000	0.580	0.705	0.000	0.000	0.000	0.000	0.000	0.258
Nonanoic_acid	2.340	0.884	4.104	1.911	3.139	5.076	2.259	1.708	7.376	1.881
Carvacrol	0.000	0.000	0.201	2.390	0.000	0.616	0.000	0.000	0.000	0.000
Ethanone_1-(2-aminophenyl)-	0.000	1.087	0.000	0.000	0.000	0.141	0.422	0.079	0.000	0.000
1H-Pyrrole-25-dione_3-ethyl-	0.137	0.209	0.220	0.295	0.122	0.290	0.300	0.343	0.302	0.191
1-Tetracosene	0.089	0.000	0.286	0.549	0.000	0.000	0.000	0.000	0.057	0.102
n-Decanoic_acid	0.323	0.176	0.080	0.000	0.227	0.314	0.000	0.000	0.069	0.320

1-Hexadecanol	1.938	0.571	4.573	9.095	0.720	1.277	0.504	0.000	0.642	2.467
(Z)6-Pentadecen-1-ol	0.243	0.000	0.239	0.230	0.000	0.000	0.000	0.000	0.153	0.376
g-Dodecalactone	0.146	0.936	1.347	0.823	0.477	0.953	0.920	0.786	0.152	0.347
Indole	8.815	9.731	11.107	10.175	5.958	5.706	17.364	8.302	20.864	8.119
1H-Indole_5-methyl-	27.987	23.896	26.118	23.388	19.170	24.772	10.305	7.165	15.437	13.504
Benzeneacetic_acid	0.152	1.148	0.000	0.000	0.000	0.000	0.000	0.000	0.115	0.000
Benzenepropanoic_acid_silver(	0.394	0.878	0.000	0.000	0.471	0.145	0.077	0.000	0.194	0.391
2H-Indol-2-one_13-dihydro-	0.115	0.428	0.138	0.118	0.140	0.206	0.213	0.106	0.121	0.067
**sample_id**	**20059_1**	**20059_2**	**20074_1**	**20074_2**	**20081_1**	**20081_2**	**20085_1**	**20085_2**	**20089_1**	**20089_2**
**group**	**ATFIS**	**ATFIS**	**ATFIS**	**ATFIS**	**ATFIS**	**ATFIS**	**ATFIS**	**ATFIS**	**ATFIS**	**ATFIS**
Methyl_acetate	0.000	0.000	0.000	0.000	0.000	0.000	0.000	0.000	0.000	0.000
Ethyl_Acetate	0.230	0.000	0.000	0.000	0.000	0.000	0.000	0.000	0.000	0.000
2-Butanone	0.018	0.206	0.574	0.199	0.098	0.107	0.009	0.059	0.039	0.102
Butanal_3-methyl-	0.405	2.060	1.322	1.033	0.607	1.263	2.272	0.964	1.878	6.538
Ethanol	0.300	0.000	0.107	0.089	0.132	0.194	0.021	0.578	0.042	0.232
Propanoic_acid_ethyl_ester	0.798	0.000	0.000	0.000	0.159	0.000	0.000	0.000	0.000	0.000
2-Pentanone	0.000	0.242	0.861	0.000	0.300	0.000	0.000	0.508	0.000	0.000
Butanoic_acid_methyl_ester	0.889	0.000	0.727	0.295	0.364	0.242	0.000	0.000	0.000	0.000
Methyl_Isobutyl_Ketone	0.140	0.159	0.466	0.443	0.369	0.278	0.097	0.107	0.067	0.152
a-Pinene	0.000	0.265	0.000	0.048	0.000	0.086	0.000	0.866	0.923	0.000
Disulfide_dimethyl	0.000	0.162	0.147	0.171	0.000	0.000	1.128	0.358	0.037	0.191
Butanoic_acid_3-methyl-_ethy	0.000	0.026	0.036	0.018	0.156	0.000	0.000	0.000	0.000	0.016
Methyl_valerate	0.590	0.053	0.458	0.316	0.200	0.218	0.000	0.000	0.000	0.000
b-Pinene	0.000	0.279	0.000	0.000	0.000	0.000	0.120	5.199	0.000	0.000
Butanoic_acid_propyl_ester	9.636	0.000	0.000	0.120	0.348	0.086	0.000	0.000	0.000	0.000
3-Carene	0.000	1.059	0.000	0.177	0.000	0.163	0.000	0.159	0.182	0.000
Pentanoic_acid_ethyl_ester	3.051	0.000	0.256	0.199	0.691	0.282	0.000	0.000	0.000	0.162
(2E4E)-37-Dimethylocta-24-d	0.000	0.000	0.502	0.392	0.000	0.106	0.000	0.216	0.000	0.000
a-Phellandrene	0.000	0.096	0.000	0.000	0.000	0.039	0.000	0.000	0.107	0.000
b-Myrcene	0.000	0.000	0.000	0.111	0.000	0.191	0.000	0.284	7.208	0.000
Isovaleric_acid_propyl_ester	0.343	0.000	0.000	0.000	0.411	0.000	0.000	0.000	0.000	0.000
Terpinolene	0.000	0.000	0.000	0.000	0.000	0.255	0.000	0.291	0.000	0.000
1-Butanol	0.218	0.130	0.000	0.000	0.390	0.138	0.000	0.026	0.000	0.000
D-Limonene	0.587	1.855	19.410	8.726	0.762	0.582	0.587	145.246	438.882	0.450
beta-Phellandrene	0.000	0.080	0.000	0.000	0.000	0.000	0.000	0.000	0.000	0.000
Butanoic_acid_butyl_ester	0.000	0.000	0.047	0.075	0.211	0.089	0.000	0.000	0.000	0.000
Pentanoic_acid_propyl_ester	6.979	0.000	0.133	0.000	0.320	0.000	0.000	0.000	0.000	0.000
g-Terpinene	0.000	0.000	0.097	0.398	0.594	1.747	0.000	9.639	0.000	0.058
Hexanoic_acid_ethyl_ester	1.597	0.000	0.554	0.443	0.997	1.171	0.000	0.000	0.000	0.000

1-Butanol_3-methyl-	0.000	0.000	0.091	0.000	0.000	0.000	0.000	0.000	0.000	0.167
b-Ocimene	0.000	0.000	0.000	0.000	0.000	0.000	0.000	0.003	0.000	0.103
Styrene	0.000	0.000	0.000	0.000	0.000	0.000	0.000	0.000	0.000	0.000
p-Cymene	0.000	0.220	0.000	0.072	0.493	1.621	0.000	0.927	0.000	0.000
2-Heptanone_5-methyl-	0.187	0.000	0.065	0.000	0.000	0.000	0.153	0.000	0.000	0.075
2-Carene	0.000	0.000	0.037	0.000	0.000	0.000	0.000	0.000	0.000	0.000
1-Pentanol	0.000	0.000	0.188	0.000	0.000	0.000	0.000	0.000	0.000	0.066
Acetic_acid_hexyl_ester	0.019	0.000	0.000	0.000	0.019	0.095	0.000	0.000	0.000	0.000
Pentanoic_acid_4-methyl-_pen	0.000	0.000	0.000	0.000	0.000	0.000	0.000	0.000	0.000	0.000
Octanal	0.000	0.000	0.000	0.000	0.000	0.000	0.000	0.000	0.000	0.169
2-Octanone	0.000	0.000	0.307	0.221	0.109	0.128	0.211	0.000	0.000	0.000
Acetoin	0.000	0.000	2.472	0.000	0.000	0.000	0.000	10.972	0.595	0.137
Pentanoic_acid_butyl_ester	2.476	0.045	0.108	0.278	0.200	0.077	0.000	0.000	0.000	0.000
Butanoic_acid_pentyl_ester	0.000	0.000	0.000	0.000	0.000	0.051	0.000	0.000	0.000	0.000
Hexanoic_acid_propyl_ester	2.141	0.000	0.208	0.159	0.304	0.184	0.000	0.000	0.000	0.000
Tridecane	0.000	0.000	0.000	0.000	0.000	0.000	0.038	0.000	0.000	0.004
5-Hepten-2-one_6-methyl-	0.577	1.367	1.043	3.240	0.394	1.380	1.430	0.000	0.395	2.152
Heptanoic_acid_ethyl_ester	0.000	0.000	0.184	0.000	0.071	0.751	0.000	0.000	0.000	0.000
Dimethyl_trisulfide	0.000	0.041	0.000	0.000	0.000	0.000	0.309	0.264	0.000	0.000
Allyl_Isothiocyanate	0.000	0.000	0.000	0.259	0.037	0.000	0.000	0.000	0.000	0.000
2-Hydroxy-3-pentanone	0.000	0.000	0.000	0.000	0.000	0.000	0.000	0.105	0.000	0.000
Hexanoic_acid_isobutyl_ester	0.000	0.000	0.000	0.000	0.000	0.000	0.000	0.000	0.000	0.000
2-butyl-1-octanol	0.009	0.038	0.000	0.000	0.000	0.039	0.903	0.000	0.024	0.348
Cyclohexanecarboxylic_acid_me	1.375	0.000	0.210	0.067	0.000	0.000	0.000	0.000	0.000	0.000
1-Hexanol	0.000	0.031	0.329	0.702	0.949	0.851	0.000	0.000	0.000	0.000
Nonanal	0.529	0.863	0.207	0.144	0.288	0.754	0.280	0.410	0.228	0.632
2-Nonanone	0.000	0.000	0.289	0.107	0.081	0.000	0.093	0.000	0.000	0.000
Cyclohexanecarboxylic_acid_et	3.387	0.000	0.041	0.000	0.024	0.000	0.000	0.000	0.014	0.000
Perillene	0.000	0.087	0.000	0.041	0.000	0.025	0.013	0.000	0.003	0.049
Pentanoic_acid_pentyl_ester	0.000	0.154	0.000	0.000	0.000	0.000	0.000	0.000	0.000	0.000
Hexanoic_acid_butyl_ester	1.054	0.000	0.200	0.518	0.347	0.277	0.000	0.000	0.000	0.000
Butanoic_acid_hexyl_ester	0.000	0.000	0.000	0.000	0.343	0.319	0.000	0.000	0.000	0.000
Tetradecane	0.136	0.876	0.482	0.339	0.206	0.363	0.000	0.000	0.000	0.682
Octanoic_acid_ethyl_ester	0.000	0.000	0.000	0.000	0.000	0.224	0.000	0.000	0.000	0.000
Acetic_acid	14.275	2.532	8.615	2.145	3.801	2.564	0.922	5.709	1.173	2.128
(+)-2-Bornanone	0.000	0.000	0.000	0.000	0.000	0.000	0.000	0.000	0.000	0.000
2-Decanone	0.000	0.000	0.000	0.000	0.000	0.000	0.000	0.000	0.000	0.000
Benzaldehyde	0.849	1.865	0.877	0.596	0.927	0.750	4.808	1.048	1.398	1.998
1-Hexanol_2-ethyl-	0.000	0.000	0.000	0.000	0.000	0.000	0.273	0.000	0.000	0.085

2-Nonenal_(E)-	0.000	0.096	0.036	0.077	0.057	0.117	0.092	0.065	0.000	0.176
Pentadecane	0.000	0.000	0.860	0.437	0.464	0.390	0.017	0.000	0.186	0.000
Propanoic_acid	18.939	3.720	4.978	2.006	3.431	1.907	1.585	2.634	0.915	2.466
Caparratriene	0.000	0.000	0.000	0.000	0.000	0.000	0.000	0.000	0.000	0.000
1-Pentadecene	0.000	0.000	0.474	0.101	0.048	0.000	0.014	0.000	0.002	0.361
Linalool	0.155	0.323	0.000	0.000	0.297	0.352	0.000	0.180	0.000	0.000
Propanoic_acid_2-methyl-	2.797	1.240	2.050	0.642	1.199	0.834	0.357	0.901	0.210	1.744
Caryophyllene	0.302	2.677	0.129	1.380	0.357	1.530	1.210	1.066	1.779	0.241
Methyl_carvacrol	0.000	0.000	0.000	0.000	0.531	0.374	0.000	0.000	0.000	0.000
Dichloroacetic_acid_4-pentade	0.000	0.000	0.000	0.000	0.000	0.000	0.000	0.000	0.000	0.000
2-Undecanone	0.214	0.738	0.915	0.697	0.493	0.506	0.678	0.370	0.291	0.692
159-Decatriene_2358-tetr	0.000	0.000	0.000	0.000	0.000	0.000	0.297	0.000	0.000	0.000
Benzeneacetaldehyde	0.000	0.000	0.000	0.000	0.000	0.000	0.000	0.299	0.345	1.005
Butanoic_acid	67.687	12.855	16.723	14.696	26.837	17.790	2.164	8.246	2.009	6.734
Hexadecane	0.133	0.270	0.262	0.233	0.208	0.283	0.472	0.171	0.075	0.256
Levomenthol	0.000	0.000	0.282	0.444	1.009	0.990	0.226	0.000	0.000	0.289
Hexanoic_acid_2-methyl-	10.680	7.676	9.884	3.861	7.867	4.975	1.698	4.836	0.876	7.983
Cetene	0.000	0.000	0.134	0.090	0.041	0.042	0.000	0.000	0.000	0.069
Valencene	0.000	0.214	1.404	0.562	0.409	0.000	0.000	0.474	49.734	0.000
a-Muurolene	0.000	0.000	0.000	0.000	0.000	0.000	0.000	0.000	0.529	0.000
b-Bisabolene	0.000	0.000	0.000	0.179	0.000	0.000	0.103	0.570	0.000	0.388
Nonadecane	0.000	0.000	0.000	0.000	0.000	0.000	0.000	0.000	0.000	0.000
Pentanoic_acid	28.656	12.887	20.864	12.891	12.662	11.992	2.566	5.091	0.682	5.684
g-Cadinene	0.000	0.000	0.000	0.000	0.000	0.000	0.000	0.000	0.000	0.000
trans-g-Bisabolene	0.000	0.397	0.000	0.075	0.000	0.722	0.000	0.000	0.157	0.504
trans-a-Bisabolene	0.000	0.088	0.000	0.135	0.000	0.000	0.000	0.000	0.000	0.399
Aromandendrene	0.000	0.000	0.000	0.000	0.000	0.000	0.000	0.000	0.000	0.000
Pentanoic_acid_4-methyl-	0.414	0.432	0.215	0.000	0.164	0.184	0.109	0.603	0.034	0.000
Germacrene_B	0.000	0.000	0.000	0.000	0.000	0.000	0.000	0.000	0.000	0.000
Anethole	0.741	0.217	0.000	0.164	1.515	0.119	0.090	0.085	0.000	0.107
2-Tridecanone	0.134	0.259	0.380	0.151	0.233	0.156	0.347	0.291	0.405	0.808
Octadecane	0.000	0.000	0.000	0.000	0.000	0.000	1.379	0.000	0.000	0.176
Hexanoic_acid	24.059	7.137	41.350	34.799	39.419	46.992	0.613	0.783	0.258	0.933
59-Undecadien-2-one_610-dim	0.420	0.286	0.475	0.383	0.000	0.000	0.189	0.050	0.175	0.155
2-Tetradecanone	0.098	0.118	0.084	0.078	0.124	0.163	0.123	0.000	0.033	0.000
Phenylethyl_Alcohol	0.387	0.450	2.283	0.626	0.373	0.000	0.074	0.114	0.175	0.375
2-Tetradecanone	0.000	0.079	0.000	0.000	0.000	0.000	0.122	0.000	0.037	0.058
Heptanoic_acid	1.938	0.783	0.370	0.221	10.677	26.467	0.235	0.235	0.000	0.284
Phenol	0.362	0.354	0.715	0.933	1.778	0.651	12.226	3.576	0.894	0.555
2-Pentadecanone	0.197	0.208	0.287	0.115	0.158	0.178	0.264	0.166	0.205	0.215
3-Phenylpropanol	0.031	0.037	0.165	0.177	0.265	0.133	0.000	0.000	0.106	0.036
Octanoic_acid	0.422	0.162	6.690	2.377	1.511	12.220	0.104	0.137	0.082	0.154
Phenol_2-methyl-	12.165	69.959	59.164	51.602	49.067	40.975	79.538	73.872	43.140	102.534
2-Hexadecanone	0.000	0.000	0.000	0.000	0.000	0.000	0.000	0.000	0.000	0.000
2-Piperidinone	1.244	0.000	0.309	0.000	0.333	0.147	0.000	0.000	0.000	0.000
2-Tridecanol	0.000	0.000	0.163	0.000	0.000	0.000	0.001	0.034	0.000	0.000

Nonanoic_acid	8.910	5.787	5.557	3.235	2.129	0.728	0.739	7.567	5.230	7.840
Carvacrol	0.000	0.000	0.000	0.139	2.058	5.861	0.000	0.000	0.000	0.000
Ethanone_1-(2-aminophenyl)-	0.000	0.000	0.000	0.000	0.000	0.270	0.182	0.000	0.000	0.702
1H-Pyrrole-25-dione_3-ethyl-	0.428	0.560	0.292	0.000	0.454	0.522	1.434	0.255	0.328	0.853
1-Tetracosene	0.000	0.020	0.151	0.078	0.000	0.000	0.000	0.219	0.042	0.105
n-Decanoic_acid	0.290	0.003	0.759	0.266	0.180	0.245	0.000	0.000	0.000	0.000
1-Hexadecanol	1.371	0.635	4.881	2.528	0.349	0.317	1.766	1.379	3.207	1.793
(Z)6-Pentadecen-1-ol	0.340	0.129	0.298	0.367	0.325	0.000	5.713	2.616	0.091	0.000
g-Dodecalactone	0.139	1.034	0.527	0.313	0.430	0.688	0.717	1.299	0.128	0.332
Indole	17.766	7.645	12.651	11.033	28.114	15.587	37.618	10.604	21.807	37.058
1H-Indole_5-methyl-	2.228	3.621	0.000	0.000	3.278	1.016	4.055	12.336	0.670	2.059
Benzeneacetic_acid	0.657	0.000	0.422	0.087	0.213	0.274	0.000	0.000	0.000	0.000
Benzenepropanoic_acid_silver(	0.697	0.095	0.946	0.347	0.664	0.847	0.000	0.247	0.130	0.000
2H-Indol-2-one_13-dihydro-	0.092	0.182	0.246	0.146	0.079	0.046	0.134	0.244	0.047	0.072
**sample_id**	**20092_1**	**20092_2**	**20095_1**	**20095_2**	**20102_1**	**20102_2**	**20104_1**	**20104_2**	**20105_1**	**20105_2**
**group**	**ATFIS**	**ATFIS**	**LGIMD-ATFIS**	**LGIMD-ATFIS**	**ATFIS**	**ATFIS**	**ATFIS**	**ATFIS**	**ATFIS**	**ATFIS**
Methyl_acetate	0.000	0.000	0.000	0.000	0.192	0.362	0.000	0.000	0.000	0.000
Ethyl_Acetate	0.000	0.215	0.000	0.000	0.286	0.336	0.000	0.000	0.000	0.000
2-Butanone	0.101	0.301	0.147	0.149	0.012	0.031	0.161	0.140	0.113	0.062
Butanal_3-methyl-	2.513	0.474	2.047	1.704	0.444	0.725	2.685	2.505	0.874	2.297
Ethanol	0.043	0.830	0.062	0.000	0.327	0.466	0.084	0.038	0.100	0.206
Propanoic_acid_ethyl_ester	0.000	0.381	0.000	0.000	0.274	0.458	0.000	0.000	0.000	0.000
2-Pentanone	0.341	0.000	0.205	0.000	0.242	0.000	0.478	0.715	0.000	0.000
Butanoic_acid_methyl_ester	0.000	0.554	0.000	0.000	1.429	0.459	0.000	0.000	0.837	0.000
Methyl_Isobutyl_Ketone	0.273	0.628	0.056	0.046	0.039	0.037	0.138	0.162	0.030	0.069
a-Pinene	0.368	0.636	0.132	12.657	0.000	0.233	0.083	0.000	0.000	0.102
Disulfide_dimethyl	0.212	0.000	1.948	0.986	0.000	0.000	0.710	0.654	0.000	0.000
Butanoic_acid_3-methyl-_ethy	0.042	0.000	0.000	0.018	0.000	0.000	0.022	0.000	0.013	0.000
Methyl_valerate	0.000	0.175	0.000	0.000	0.000	0.000	0.000	0.000	0.166	0.000
b-Pinene	0.000	0.201	0.000	6.365	0.000	0.000	0.303	0.000	0.000	0.312
Butanoic_acid_propyl_ester	0.000	2.139	0.000	0.000	1.182	0.384	0.000	0.000	0.213	0.000
3-Carene	0.000	0.504	0.000	0.487	0.000	0.000	0.000	0.000	0.000	0.105
Pentanoic_acid_ethyl_ester	0.000	1.080	0.000	0.000	0.166	0.088	0.000	0.000	0.000	0.000
(2E4E)-37-Dimethylocta-24-d	0.613	2.103	0.956	0.706	0.000	0.286	0.495	0.518	0.000	0.000
a-Phellandrene	0.000	0.427	0.000	0.808	0.000	0.169	0.000	0.000	0.000	0.000
b-Myrcene	0.000	1.130	0.000	2.764	0.000	0.404	0.000	0.000	0.000	0.000
Isovaleric_acid_propyl_ester	0.000	0.000	0.000	0.000	0.072	0.000	0.000	0.000	0.000	0.000
Terpinolene	0.000	0.000	0.000	1.100	0.000	0.168	0.000	0.000	0.000	0.000
1-Butanol	0.010	0.000	0.000	0.079	1.239	1.109	0.000	0.000	0.836	0.000

D-Limonene	1.599	0.000	23.574	18.756	0.000	0.766	4.231	1.216	0.000	6.926
beta-Phellandrene	0.000	0.000	0.000	8.157	0.000	0.000	0.000	0.000	0.000	0.000
Butanoic_acid_butyl_ester	0.037	2.418	0.000	0.000	1.336	0.037	0.000	0.000	0.281	0.000
Pentanoic_acid_propyl_ester	0.000	1.444	0.000	0.000	0.000	0.000	0.000	0.000	0.150	0.000
g-Terpinene	0.000	0.000	0.302	2.154	0.000	0.000	0.588	0.000	0.000	0.953
Hexanoic_acid_ethyl_ester	0.000	0.000	0.000	0.156	0.000	0.000	0.094	0.000	0.000	0.000
1-Butanol_3-methyl-	0.000	0.263	0.433	0.000	0.000	0.079	0.000	0.000	0.057	0.101
b-Ocimene	0.000	0.000	0.000	0.000	0.000	0.000	0.000	0.000	0.000	0.000
Styrene	0.000	0.000	0.000	0.000	0.000	0.000	0.066	0.015	0.030	0.247
p-Cymene	0.000	0.192	0.029	0.732	0.000	0.000	0.078	0.000	0.000	0.163
2-Heptanone_5-methyl-	0.000	0.000	0.000	0.000	0.000	0.000	0.000	0.054	0.000	0.083
2-Carene	0.000	0.334	0.092	0.012	0.000	0.141	0.102	0.228	0.000	0.000
1-Pentanol	0.000	0.434	0.096	0.258	0.000	0.063	0.109	0.035	0.383	0.159
Acetic_acid_hexyl_ester	0.000	0.000	0.000	0.000	0.000	0.000	0.000	0.000	0.000	0.000
Pentanoic_acid_4-methyl-_pen	0.000	0.131	0.000	0.000	0.000	0.000	0.000	0.000	0.000	0.000
Octanal	0.000	0.154	0.000	0.000	0.000	0.076	0.000	0.000	0.088	0.113
2-Octanone	0.000	0.000	0.165	0.283	0.000	0.000	0.000	0.330	0.000	0.121
Acetoin	0.038	0.000	0.127	0.000	0.000	0.000	0.000	0.000	0.000	0.066
Pentanoic_acid_butyl_ester	0.022	1.573	0.000	0.044	0.058	0.000	0.000	0.000	0.163	0.000
Butanoic_acid_pentyl_ester	0.031	0.000	0.000	0.000	0.000	0.000	0.000	0.000	0.000	0.000
Hexanoic_acid_propyl_ester	0.000	0.000	0.000	0.000	0.000	0.000	0.000	0.000	0.000	0.000
Tridecane	0.007	0.000	0.073	0.110	0.000	0.000	0.027	0.000	0.082	0.005
5-Hepten-2-one_6-methyl-	8.266	2.391	1.028	6.194	0.167	0.498	2.953	1.458	0.619	3.059
Heptanoic_acid_ethyl_ester	0.000	0.000	0.000	0.000	0.000	0.000	0.000	0.000	0.000	0.000
Dimethyl_trisulfide	0.000	0.000	0.922	0.597	0.000	0.000	0.266	0.113	0.000	0.000
Allyl_Isothiocyanate	0.000	1.012	0.000	0.000	0.000	0.000	0.000	0.000	0.000	0.000
2-Hydroxy-3-pentanone	0.000	0.000	0.061	0.000	0.000	0.000	0.100	0.075	0.000	0.000
Hexanoic_acid_isobutyl_ester	0.000	0.000	0.000	0.000	0.000	0.000	0.000	0.000	0.000	0.000
2-butyl-1-octanol	0.000	0.000	0.000	0.282	0.000	0.000	0.000	0.008	0.199	0.010
Cyclohexanecarboxylic_acid_me	0.000	0.532	0.000	0.000	5.052	34.323	0.000	0.000	0.407	0.000
1-Hexanol	0.024	0.409	0.214	0.789	0.000	0.000	0.253	0.134	0.000	0.000
Nonanal	0.152	0.723	0.324	0.277	0.736	0.610	0.347	0.338	0.928	0.965
2-Nonanone	0.000	0.000	0.000	0.069	0.000	0.000	0.052	0.124	0.000	0.196
Cyclohexanecarboxylic_acid_et	0.000	1.311	0.000	0.000	7.148	39.620	0.045	0.000	0.000	0.000
Perillene	0.001	0.000	0.011	0.081	0.000	0.000	0.031	0.010	0.000	0.000
Pentanoic_acid_pentyl_ester	0.000	0.236	0.000	0.000	0.000	0.000	0.000	0.000	0.000	0.000
Hexanoic_acid_butyl_ester	0.000	0.000	0.000	0.161	0.000	0.000	0.000	0.000	0.000	0.000
Butanoic_acid_hexyl_ester	0.000	0.151	0.000	0.111	0.000	0.000	0.000	0.000	0.000	0.000

Tetradecane	0.807	0.358	0.235	1.181	0.059	0.095	0.724	0.428	0.425	0.355
Octanoic_acid_ethyl_ester	0.000	0.000	0.000	0.000	0.000	0.000	0.000	0.000	0.000	0.000
Acetic_acid	2.503	13.491	1.862	2.993	4.143	13.467	2.914	1.682	7.433	3.492
(+)-2-Bornanone	0.000	0.000	0.000	5.603	0.000	0.000	0.000	0.000	0.000	0.000
2-Decanone	0.000	0.000	0.000	0.000	0.000	0.000	0.000	0.000	0.011	0.000
Benzaldehyde	1.729	4.756	1.799	1.146	0.399	1.957	1.657	2.557	0.808	3.190
1-Hexanol_2-ethyl-	0.000	0.000	0.000	0.000	0.000	0.000	0.000	0.000	0.000	0.152
2-Nonenal_(E)-	0.038	0.199	0.091	0.000	0.353	0.089	0.116	0.079	0.228	0.075
Pentadecane	0.476	0.000	1.956	0.000	0.000	0.000	0.000	0.471	0.000	0.027
Propanoic_acid	1.907	8.458	22.463	2.668	2.171	6.350	2.834	0.994	2.874	1.750
Caparratriene	0.000	0.000	0.000	0.000	0.000	0.000	0.000	0.000	0.000	0.000
1-Pentadecene	0.184	0.000	0.602	0.286	0.000	0.000	0.167	0.221	0.004	0.000
Linalool	0.000	2.205	0.000	0.760	0.392	0.420	0.000	0.000	0.507	0.000
Propanoic_acid_2-methyl-	1.957	0.945	1.004	1.924	0.081	0.426	1.789	1.024	1.131	0.411
Caryophyllene	0.369	1.979	1.237	34.348	0.183	0.448	0.579	0.740	0.554	1.567
Methyl_carvacrol	0.000	0.041	0.000	0.000	0.000	0.000	0.000	0.000	0.002	0.000
Dichloroacetic_acid_4-pentade	0.000	0.000	0.000	0.000	0.000	0.000	0.000	0.000	0.000	0.000
2-Undecanone	0.497	0.815	0.488	1.237	0.540	0.278	0.592	0.678	0.329	0.658
159-Decatriene_2358-tetr	0.000	0.000	0.697	0.238	0.000	0.000	0.000	0.000	0.000	0.000
Benzeneacetaldehyde	0.260	0.000	0.000	0.000	0.000	0.000	0.624	0.519	0.000	0.000
Butanoic_acid	16.740	52.060	4.158	9.001	45.989	50.256	8.740	4.563	30.808	13.719
Hexadecane	0.205	0.000	0.743	0.457	0.000	0.000	0.309	0.376	0.394	0.528
Levomenthol	0.369	2.110	0.000	0.000	2.765	0.000	0.000	0.000	1.024	1.082
Hexanoic_acid_2-methyl-	11.190	5.145	5.098	10.108	1.075	1.819	7.724	5.088	4.115	1.179
Cetene	0.150	0.000	0.125	0.084	0.000	0.000	0.086	0.215	0.000	0.002
Valencene	0.000	1.809	2.374	1.760	1.599	2.413	0.000	0.000	0.000	0.037
a-Muurolene	0.000	0.000	0.210	0.169	0.000	0.000	0.000	0.000	0.000	0.000
b-Bisabolene	0.070	2.024	0.000	0.490	0.000	0.000	0.318	0.000	0.000	0.508
Nonadecane	0.000	0.000	0.000	0.000	0.000	0.000	0.085	0.000	0.000	0.000
Pentanoic_acid	9.057	15.849	4.648	8.846	1.004	2.371	6.394	4.165	7.102	2.205
g-Cadinene	0.000	0.000	0.267	0.623	0.000	0.000	0.000	0.000	0.000	0.304
trans-g-Bisabolene	0.000	27.620	0.000	0.000	0.000	2.620	0.392	0.323	0.743	0.000
trans-a-Bisabolene	0.000	0.112	0.000	0.000	0.000	0.000	0.038	0.000	0.000	0.000
Aromandendrene	0.084	1.182	0.078	0.000	0.000	1.113	0.164	0.252	0.000	0.000
Pentanoic_acid_4-methyl-	1.934	2.239	0.109	0.192	0.407	0.254	0.185	0.221	0.650	0.526
Germacrene_B	0.000	0.000	0.000	0.667	0.000	0.000	0.000	0.000	0.000	0.000
Anethole	0.111	0.516	0.662	0.073	0.000	0.000	0.581	1.195	0.046	0.152
2-Tridecanone	0.347	0.340	0.384	0.514	0.086	0.000	0.141	0.332	0.185	0.425
Octadecane	0.134	0.094	2.179	0.000	0.000	0.000	0.000	0.000	0.000	2.656
Hexanoic_acid	2.692	2.028	7.345	19.099	0.627	0.286	12.948	10.615	0.983	0.415
59-Undecadien-2-one_610-dim	0.180	0.000	0.244	0.418	0.000	0.067	0.269	0.264	0.089	0.141
2-Tetradecanone	0.097	0.000	0.119	0.000	0.000	0.000	0.000	0.077	0.000	0.109
Phenylethyl_Alcohol	0.764	0.685	0.000	0.000	0.000	0.088	0.562	0.692	0.080	0.305
2-Tetradecanone	0.113	0.000	0.094	0.000	0.000	0.000	0.000	0.065	0.000	0.000
Heptanoic_acid	0.207	0.000	4.362	10.907	0.092	0.108	4.504	7.603	0.115	0.000

Phenol	0.237	0.665	0.176	0.248	0.522	0.420	1.117	1.272	1.199	8.255
2-Pentadecanone	0.275	0.183	0.308	0.281	0.116	0.132	0.196	0.174	0.218	0.252
3-Phenylpropanol	0.143	0.099	0.000	0.000	0.000	0.000	0.000	0.046	0.001	0.001
Octanoic_acid	0.140	0.511	0.985	2.203	4.847	0.016	0.987	2.030	0.148	0.178
Phenol_2-methyl-	69.849	11.547	47.433	59.173	26.927	154.373	90.477	59.222	37.020	32.753
2-Hexadecanone	0.000	0.000	0.000	0.000	0.000	0.000	0.000	0.000	0.000	0.000
2-Piperidinone	0.000	0.000	0.000	0.000	0.000	0.000	0.000	0.000	0.000	0.128
2-Tridecanol	0.000	0.000	0.000	0.000	0.000	0.000	0.000	0.000	0.000	0.000
Nonanoic_acid	2.215	0.743	5.290	5.408	5.916	2.798	10.845	2.026	0.899	6.177
Carvacrol	0.000	0.325	0.000	0.000	0.000	0.000	0.000	0.000	0.411	0.000
Ethanone_1-(2-aminophenyl)-	0.000	0.000	0.000	0.000	0.000	0.000	0.000	0.000	0.000	0.158
1H-Pyrrole-25-dione_3-ethyl-	0.268	0.361	0.169	0.243	0.109	0.931	0.325	0.254	1.207	0.707
1-Tetracosene	0.234	0.051	0.000	0.000	0.000	0.000	0.104	0.130	0.000	0.000
n-Decanoic_acid	0.104	0.164	0.287	0.314	1.115	0.000	0.178	0.066	0.000	0.000
1-Hexadecanol	1.545	1.270	0.256	0.147	0.031	0.000	1.055	1.869	0.697	0.377
(Z)6-Pentadecen-1-ol	0.000	0.891	0.000	0.000	0.000	0.000	0.151	0.252	1.356	0.133
g-Dodecalactone	0.784	1.215	0.338	0.365	0.189	0.468	0.809	0.237	0.936	0.142
Indole	13.946	15.570	15.374	16.829	8.052	4.271	20.094	7.930	19.018	15.362
1H-Indole_5-methyl-	16.314	2.309	6.396	7.301	0.421	0.670	16.063	0.000	7.395	0.548
Benzeneacetic_acid	0.184	0.000	0.000	0.189	0.000	0.000	0.000	0.000	0.000	0.000
Benzenepropanoic_acid_silver(	0.080	0.575	0.065	0.213	0.000	0.000	0.000	0.126	0.220	0.000
2H-Indol-2-one_13-dihydro-	0.048	0.000	0.141	0.266	0.043	0.000	0.108	0.097	0.100	0.037

**sample_id**	**20121_1**	**20121_2**	**20123_1**	**20123_2**	**20124_1**	**20124_2**
**group**	**ATFIS**	**ATFIS**	**ATFIS**	**ATFIS**	**ATFIS**	**ATFIS**
Methyl_acetate	0.000	0.000	0.000	0.000	0.000	0.000
Ethyl_Acetate	0.000	0.000	0.000	0.000	0.000	0.000
2-Butanone	0.339	0.192	0.044	0.054	0.103	0.092
Butanal_3-methyl-	2.067	0.835	0.521	0.996	1.051	3.071
Ethanol	0.071	0.062	0.292	0.159	0.056	0.048
Propanoic_acid_ethyl_ester	0.000	0.000	0.449	0.000	0.000	0.000
2-Pentanone	0.489	0.000	0.000	0.406	0.176	0.000
Butanoic_acid_methyl_ester	0.000	0.323	1.930	0.268	0.000	0.000
Methyl_Isobutyl_Ketone	0.306	0.198	0.035	0.038	0.065	0.058
a-Pinene	0.470	0.101	0.000	0.000	0.000	0.000
Disulfide_dimethyl	0.101	0.110	0.000	0.115	0.207	0.356
Butanoic_acid_3-methyl-_ethy	0.000	0.000	0.048	0.031	0.000	0.000
Methyl_valerate	0.000	0.113	0.879	0.089	0.000	0.000
b-Pinene	0.475	0.683	0.000	0.000	0.000	0.000
Butanoic_acid_propyl_ester	0.000	0.843	0.988	0.168	0.000	0.000
3-Carene	0.000	0.000	0.098	0.000	0.000	0.000
Pentanoic_acid_ethyl_ester	0.000	0.172	1.041	0.132	0.000	0.000

(2E4E)-37-Dimethylocta-24-d	0.000	0.000	0.000	0.424	0.300	0.469
a-Phellandrene	0.000	0.000	0.000	0.000	0.000	0.000
b-Myrcene	0.054	0.160	0.000	0.000	0.000	0.000
Isovaleric_acid_propyl_ester	0.000	0.000	0.268	0.092	0.000	0.000
Terpinolene	0.048	0.000	0.000	0.000	0.000	0.000
1-Butanol	0.000	0.000	1.261	0.001	0.000	0.286
D-Limonene	9.192	10.764	0.446	0.000	1.461	0.278
beta-Phellandrene	0.140	0.000	0.000	0.000	0.000	0.000
Butanoic_acid_butyl_ester	0.000	1.079	0.485	0.187	0.000	0.000
Pentanoic_acid_propyl_ester	0.000	0.000	0.854	0.134	0.000	0.000
g-Terpinene	2.491	0.960	0.000	0.000	0.000	0.000
Hexanoic_acid_ethyl_ester	0.103	0.568	1.215	0.278	0.020	0.000
1-Butanol_3-methyl-	0.000	0.000	0.000	0.000	0.000	0.469
b-Ocimene	0.000	0.000	0.000	0.000	0.000	0.000
Styrene	0.000	0.000	0.000	0.089	0.000	0.000
p-Cymene	0.232	0.090	0.000	0.000	0.000	0.000
2-Heptanone_5-methyl-	0.000	0.000	0.288	0.345	0.000	0.000
2-Carene	0.000	0.034	0.179	0.000	0.000	0.317
1-Pentanol	0.000	0.117	0.646	0.565	0.035	0.083
Acetic_acid_hexyl_ester	0.000	0.067	0.000	0.000	0.000	0.000
Pentanoic_acid_4-methyl-_pen	0.000	0.000	0.199	0.020	0.000	0.000
Octanal	0.000	0.155	0.000	0.000	0.000	0.109
2-Octanone	0.183	0.000	0.411	0.000	0.117	0.000
Acetoin	0.000	0.000	0.000	2.395	0.000	0.146
Pentanoic_acid_butyl_ester	0.000	0.235	0.759	0.089	0.000	0.000
Butanoic_acid_pentyl_ester	0.000	0.237	0.000	0.080	0.000	0.000
Hexanoic_acid_propyl_ester	0.000	0.526	0.571	0.293	0.000	0.000
Tridecane	0.000	0.018	0.000	0.000	0.044	0.061
5-Hepten-2-one_6-methyl-	1.823	0.481	0.079	2.065	1.208	1.173
Heptanoic_acid_ethyl_ester	0.000	0.084	0.116	0.000	0.000	0.000
Dimethyl_trisulfide	0.000	0.000	0.000	0.000	0.079	0.076
Allyl_Isothiocyanate	0.000	0.000	0.000	0.000	0.000	0.000
2-Hydroxy-3-pentanone	0.000	0.000	0.000	0.000	0.000	0.087
Hexanoic_acid_isobutyl_ester	0.000	0.049	0.000	0.000	0.000	0.000
2-butyl-1-octanol	0.138	0.000	0.000	0.000	0.013	0.008
Cyclohexanecarboxylic_acid_me	0.000	0.000	0.455	0.000	0.000	0.000
1-Hexanol	0.000	0.450	1.296	1.941	0.148	0.289
Nonanal	0.116	0.703	0.501	0.558	0.325	0.552
2-Nonanone	0.000	0.000	0.000	0.089	0.000	0.000

Cyclohexanecarboxylic_acid_et	0.000	0.000	0.449	0.000	0.000	0.000
Perillene	0.037	0.000	0.000	0.039	0.000	0.030
Pentanoic_acid_pentyl_ester	0.000	0.000	0.000	0.000	0.000	0.000
Hexanoic_acid_butyl_ester	0.000	0.523	0.638	0.000	0.000	0.000
Butanoic_acid_hexyl_ester	0.000	1.213	0.248	0.577	0.000	0.000
Tetradecane	1.002	0.325	0.086	0.000	0.144	0.635
Octanoic_acid_ethyl_ester	0.000	0.000	0.000	0.000	0.000	0.000
Acetic_acid	4.151	6.339	9.645	8.642	8.368	4.570
(+)-2-Bornanone	0.000	0.000	0.000	0.000	0.000	0.000
2-Decanone	0.022	0.000	0.000	0.000	0.000	0.000
Benzaldehyde	0.940	0.587	0.637	0.794	0.366	2.017
1-Hexanol_2-ethyl-	0.000	0.000	0.000	0.000	0.000	0.000
2-Nonenal_(E)-	0.015	0.219	0.237	0.000	0.200	0.074
Pentadecane	0.758	0.000	0.000	0.000	0.000	0.000
Propanoic_acid	1.583	3.724	9.118	8.807	4.025	2.124
Caparratriene	0.000	0.000	0.000	0.000	0.000	0.000
1-Pentadecene	0.408	0.064	0.000	0.552	0.280	0.307
Linalool	0.000	0.000	0.128	0.000	0.000	0.000
Propanoic_acid_2-methyl-	1.540	1.364	1.020	2.545	2.996	1.035
Caryophyllene	1.089	0.268	3.103	0.000	0.300	1.498
Methyl_carvacrol	0.000	0.000	0.000	0.000	0.000	0.000
Dichloroacetic_acid_4-pentade	0.169	0.000	0.002	0.144	0.109	0.000
2-Undecanone	0.580	0.198	0.369	0.337	0.196	0.430
159-Decatriene_2358-tetr	0.479	0.000	0.000	0.000	0.000	0.000
Benzeneacetaldehyde	0.000	0.000	0.000	0.000	0.000	0.501
Butanoic_acid	7.714	22.550	35.748	47.662	14.384	7.611
Hexadecane	0.460	0.000	0.000	0.192	0.210	0.331
Levomenthol	0.133	0.179	0.000	0.000	0.000	0.344
Hexanoic_acid_2-methyl-	7.996	5.740	3.531	9.104	7.493	4.706
Cetene	0.142	0.015	0.000	0.269	0.066	0.000
Valencene	1.188	0.146	0.000	0.270	1.220	2.878
a-Muurolene	0.000	0.000	0.000	0.000	0.000	0.000
b-Bisabolene	0.305	0.127	0.000	0.000	0.000	0.000
Nonadecane	0.000	0.000	0.176	0.466	0.000	0.000
Pentanoic_acid	6.184	7.472	16.446	28.087	6.158	3.832
g-Cadinene	0.000	0.000	0.000	0.000	0.000	0.000
trans-g-Bisabolene	2.414	0.000	1.738	0.000	0.000	0.000
trans-a-Bisabolene	0.000	0.000	0.273	0.000	0.000	0.000
Aromandendrene	0.262	0.111	0.045	0.142	0.113	0.000
Pentanoic_acid_4-methyl-	0.000	0.081	1.544	4.870	0.145	0.153
Germacrene_B	0.000	0.000	0.000	0.000	0.000	0.000
Anethole	0.247	0.044	0.306	0.048	0.127	0.000
2-Tridecanone	0.481	0.103	0.342	0.364	0.292	0.355

Octadecane	0.000	0.000	0.000	0.000	0.000	0.000
Hexanoic_acid	6.794	21.310	35.953	76.418	11.985	8.194
59-Undecadien-2-one_610-dim	0.000	0.312	0.394	0.238	0.185	0.202
2-Tetradecanone	0.000	0.000	0.269	0.313	0.143	0.338
Phenylethyl_Alcohol	0.376	0.146	0.000	0.242	0.216	0.450
2-Tetradecanone	0.110	0.000	0.000	0.000	0.187	0.154
Heptanoic_acid	2.093	3.933	14.746	35.855	3.570	2.954
Phenol	0.394	0.320	4.182	3.350	0.242	0.345
2-Pentadecanone	0.475	0.110	0.276	0.349	0.461	0.732
3-Phenylpropanol	0.066	0.070	0.000	0.004	0.000	0.013
Octanoic_acid	0.279	0.531	0.930	6.926	0.923	0.297
Phenol_2-methyl-	95.079	46.707	1.895	29.816	73.469	69.530
2-Hexadecanone	0.000	0.000	0.000	0.000	0.000	0.084
2-Piperidinone	0.404	0.228	0.000	0.000	0.000	0.000
2-Tridecanol	0.207	0.000	0.001	0.029	0.001	0.200
Nonanoic_acid	2.216	0.473	0.474	14.600	4.281	6.372
Carvacrol	0.000	0.000	0.000	0.000	0.000	0.000
Ethanone_1-(2-aminophenyl)-	0.778	0.000	0.000	0.000	0.140	0.121
1H-Pyrrole-25-dione_3	0.387	0.126	0.428	0.182	0.652	1.332
1-Tetracosene	0.309	0.000	0.000	0.000	0.000	0.969
n-Decanoic_acid	0.194	0.000	0.074	0.458	0.000	0.000
1-Hexadecanol	2.233	0.363	0.462	2.334	1.200	2.664
(Z)6-Pentadecen-1-ol	0.070	0.000	1.491	1.620	0.000	0.663
g-Dodecalactone	0.564	0.633	0.593	0.350	0.538	0.274
Indole	15.666	12.457	3.897	5.969	12.462	25.892
1H-Indole_5-methyl-	0.000	7.104	6.399	18.480	21.288	29.156
Benzeneacetic_acid	0.354	0.223	0.000	0.000	0.000	0.000
Benzenepropanoic_acid_silver(	0.393	0.262	0.835	1.378	1.225	0.289
2H-Indol-2-one_13-dihydro-	0.322	0.173	0.055	0.100	0.292	0.216

Table S2. Statistically significant different VOCs in the two sample groups. Statistically significant VOC emerged from fold change (FC) and Wilcoxon rank-sum test combined analysis. For each variable fold change, log2(FC), the raw p-value (reported as raw p-value) and –log10(p) i.e. the level of significance have been reported.

VOC	FC	log2(FC)	raw.pval	-log10(p)
**5-Hepten-2-one_6-methyl-**	0.13801	-2.8571	5.9172E-06	5.2279
**Methyl_valerate**	7.254	2.8588	0.0012099	2.9173
**Butanoic_acid_methyl_ester**	6.7258	2.7497	0.0022201	2.6536
**2-Piperidinone**	5.0665	2.341	0.0045542	2.3416
**Cetene**	0.27024	-1.8877	0.0054196	2.266
**Perillene**	0.14852	-2.7513	0.0075755	2.1206
**1-Pentadecene**	0.26017	-1.9425	0.008292	2.0813
**Pentanoic_acid_ethyl_ester**	7.1731	2.8426	0.0095004	2.0223
**Levomenthol**	2.5802	1.3675	0.021809	1.6614
**Dimethyl_trisulfide**	0.46854	-1.0938	0.024503	1.6108
**Butanoic_acid_butyl_ester**	7.681	2.9413	0.026669	1.574
**Anethole**	0.21297	-2.2313	0.029753	1.5265
**Butanoic_acid_propyl_ester**	17.426	4.1232	0.029825	1.5254
**1-Butanol_3-methyl-**	0.45345	-1.141	0.037255	1.4288
**Tridecane**	0.39358	-1.3453	0.041799	1.3788
**Pentanoic_acid_butyl_ester**	8.3522	3.0622	0.043704	1.3595
**159-Decatriene_2358-tetr**	0.27422	-1.8666	0.044854	1.3482
**Heptanoic_acid_ethyl_ester**	2.2212	1.1513	0.046029	1.337

Table S3. MaAsLin supporting statistics. Statistically significant taxa ($p<0.05$) plus coef=effect estimate coefficient, stderr=standard error, N=number of sample, N.not.0=number of sample value different from 0, pval=p value, qval=corrected p.

Taxa	value	coef	stderr	N	N.not.0	pval	qval
**f__Peptococcaceae_g__uncultured**	LGMID_ATFIS	0.4738	0.1130	46	19	0.0001	0.0206
**o__Bacteroidales;f__;g__**	LGMID_ATFIS	0.7964	0.2357	46	39	0.0015	0.0344
**g__Rikenellaceae_RC9_gut_group**	LGMID_ATFIS	0.6065	0.1763	46	8	0.0013	0.0344
**g__Catenibacterium**	LGMID_ATFIS	0.2717	0.0804	46	8	0.0015	0.0344
**g__UCG2**	LGMID_ATFIS	0.5843	0.1704	46	45	0.0013	0.0344
**g__UCG10**	LGMID_ATFIS	0.8424	0.2431	46	34	0.0012	0.0344
**g__Family_XIII_AD3011_group**	LGMID_ATFIS	0.5263	0.1527	46	36	0.0013	0.0344
**g__GCA900066575**	LGMID_ATFIS	0.4147	0.1271	46	32	0.0021	0.0420
**g__Haemophilus**	LGMID_ATFIS	-0.6818	0.2123	46	23	0.0025	0.0432
**g__Sanguibacteroides**	LGMID_ATFIS	0.3629	0.1236	46	16	0.0053	0.0585
**g__Erysipelotrichaceae_UCG3**	LGMID_ATFIS	-0.4867	0.1679	46	43	0.0058	0.0585
**g__RF39**	LGMID_ATFIS	0.6212	0.2054	46	17	0.0041	0.0585
**g__Clostridia_vadinBB60_group**	LGMID_ATFIS	0.7399	0.2560	46	37	0.0060	0.0585
**g__Dorea**	LGMID_ATFIS	0.3317	0.1135	46	44	0.0055	0.0585
**g__Tyzzerella**	LGMID_ATFIS	0.4570	0.1547	46	20	0.0050	0.0585
**g__.Eubacterium._hallii_group**	LGMID_ATFIS	0.2858	0.0966	46	44	0.0050	0.0585
